# Implication of the HLA-DQA1, HLA-DQB1 and CTLA-4 alleles in the susceptibility to type 1 diabetes in Jordanian population

**DOI:** 10.1007/s11033-025-10438-x

**Published:** 2025-03-21

**Authors:** Monther Hussain Radi Obaied, Nazmi Ozer, Hussein Ibrahim Faleh Alawneh, Ozlem Dalmizrak

**Affiliations:** 1 MedLabs Consultancy Group, Amman-Zahran St. Bldg. 130, Amman, Jordan; 2 Department of Biochemistry, Faculty of Pharmacy, Girne American University, Kyrenia, TRNC, Mersin 10, 99428 Turkey; 3 Division of Pediatric Endocrinology, Queen Rania Hospital for Children, KHMC, Amman, Jordan; 4 Department of Medical Biochemistry, Faculty of Medicine, Near East University, Nicosia, TRNC, Mersin 10, 99138 Turkey

**Keywords:** Type one diabetes mellitus, HLA-DQA1, HLA-DQB1, CTLA-4

## Abstract

**Background:**

Type 1 diabetes mellitus (T1D) is a chronic autoimmune disease caused by the selective destruction of pancreatic beta cells, leading to insulin deficiency. Both genetic and environmental factors contribute to disease susceptibility. Among genetic factors, human leukocyte antigen (HLA) class II molecules, particulary DQA1 and DQB1 haplotypes, have been associated with T1D risk. This study aimed to identify haplotypes that increase susceptibility to or provide protection against T1D in Jordanian population.

**Methods:**

A total of 200 healthy individuals and 200 T1D patients were included in the study. Genomic DNA was extracted from blood samples and HLA-DQA1, HLA-DQB1 and cytotoxic T-lymphocyte associated protein 4 (CTLA-4) gene regions were amplified by PCR. The PCR products were then subjected to restriction enzyme digestion and analyzed through agarose gel electrophoresis to determine different haplotypes.

**Results:**

Among the analyzed haplotypes, HLA-DQA1*01:01 was found to be significantly associated with increased susceptibility to T1D. In contrast, HLA-DQA1*02:01 and HLA-DQB1*05:01 appeared to provide protective effects against T1D. No significant differences were observed for other haplotypes between the control and patient groups. Additionally, no significant difference has been observed in terms of CTLA-4 polymorphisms.

**Conclusion:**

These findings suggest that HLA-DQA1*01:01 may serve as a genetic marker for T1D susceptibility, while HLA-DQA1*02:01 and HLA-DQB1*05:01 may confer protectionin the Jordanian population. Identifying these genetic risk factors could contribute to early disease prevention strategies and advanced research into additional genetic markers associated with T1D.

**Supplementary Information:**

The online version contains supplementary material available at 10.1007/s11033-025-10438-x.

## Introduction

Diabetes mellitus is a condition that impairs the ability of the body to produce or utilize insulin, resulting in elevated blood glucose levels, known as hyperglycemia [1]. In 1997, the American Diabetes Association catagorized Type 1 Diabetes Mellitus (T1D) into two subtypes: Type 1A, or immune-mediated diabetes, and idiopathic type 1 diabetes. Immune-mediated diabetes accounts for 70–90% of all cases and is characterized by the autoimmune destruction of pancreatic β-cells, primarily driven by T cells. Key indicators of β-cell destruction include glutamic acid decarboxylase 65 kDa isoform (GAD65) autoantibodies, islet cell autoantibodies (IA2), and zinc transporter 8 (ZnT8) autoantibodies. Due to the extensive damage of insulin-producing β-cells, insulin secretion is either absent or significantly reduced, leading to undetectable levels of plasma C-peptide. Idiopathic type 1 diabetes, on the other hand, is relatively uncommon (affecting 1–6% of pediatric diabetes cases) and is distinguished by the lack of autoimmune markers [2]. The development of T1D is influenced by multiple factors, some of which remain unidentified. However, it is well established that both genetic and environmental factors contribute to its onset, with genetic predisposition playing a significant role in most cases [3]. Studies have revealed a link between T1D and human leukocyte antigen (HLA) gene, located on chromosome 6. HLA gene is divided into three groups; class I, class II and class III. HLA class I encodes HLA-A, HLA-B, and HLA-C while HLA class II contains the DQA, DQB, DRA and DRB genes. The class III region has genes involved in immune functions such as tumor necrosis factor, components of the complement system, heat shock protein [4]. Given that HLA exhibits multiple haplotypes, researchers initially focused on identifying the haplotype linked to T1D. DR haplotype was found to be responsible for the genetic aspect of the disease [5]. More recent studies have revealed that DQA and DQB haplotypes contribute to susceptibility in over 50% of the diabetic cases [6].

Some studies suggest that certain non-HLA genes, such as cytotoxic T-lymphocyte associated protein 4 (CTLA-4), interferon induced with helicase C domain 1 (IFIH1), lymphoid protein tyrosine phosphatase, non-receptor type 22 (PTPN22), insulin (INS), vitamin D receptor (VDR), interleukin 2 receptor alpha (IL2RA), KIAA0350 and phosphotyrosine protein phosphatase, non-receptor 2 (PTPN2), may be associated with T1D [3].

The CTLA-4 gene is located on chromosome 2q33 [7] and encodes a T-cell receptor that regulates T-cell proliferation and apoptosis. The presentation of antigens triggers the expression of T lymphocyte specific receptor protein. As a result, CTLA-4 is strongly associated with immune and autoimmune responses and may play a role in T-cell mediated autoimmune process. A polymorphism involving an A/G substitution has been identified at position 49 in the first exon of the CTLA-4 gene [8]. Research has shown that individuals with the A/A genotype had a more inhibitory effect on T-cell proliferation compared to those with the G/G genotype. Consequently, the presence of the G allele has been linked to increased production of messenger RNA and proteins essential for T cell proliferation [9].

Research has shown that genes alone may not be sufficient to cause T1D; environmental risk factors also contribute to the development of the disease [10]. Factors such as viral infections, childhood illnesses, diet and gestational events can trigger T1D in individuals genetic predisposition [1, 11]. Extensive scientific studies highlight the significance of non-genetic factors in the progression of the disease. It is now widely accepted that environmental factors interact with genetic susceptibility, ultimately initiating an autoimmune response against pancreatic beta cells [10].

The multiple haplotypes of the HLA-DQA and DQB genes present both advantages and challenges: while they allow for a more precise identification of the genetic causes of T1D, their complexity necessitates further research, particularly considering the geographical distribution of these haplotypes. In Arab countries, where endogamous and consanguineous marriages (ranging from 10 to 70%) as well as first-cousin marriages are prevalent, T1D rates are also high. This makes the Jordanian population an ideal group for studying HLA class II haplotypes [6]. A study by Ajlouni et al., indicated a steady increase in T1D incidence in Jordan over the years. The report rate was 2.8 per 100,000 in 1992, rising to approximately 3.2 per 100,000 in 1994, and reaching 3.6 per 100,000 in 1996. The highest incidence was observed among individuals aged 10 to 14 (3.2 per 100,000), while the lowest was found in children under 4 years old (1.3 per 100,000) [12]. Although global research on diabetes risk factors is extensive, studies specific to Jordan remain limited. Considering the genetic background, lifestyle and dietary habits of Jordanians, identifying population-specific risk factors could enhance prevention and intervention strategies. Since diabetes places a significant burden on the healthcare system, recognizing these risk factors may help reduce hospitalizations and diabetes-related complications. Therefore, this study aims to investigate the association between HLA-DQA1, HLA-DQB1, and CTLA-4 haplotypes with T1D in Jordanian population.

## Materials and methods

### Study population

A total of 200 (whole blood) control samples (96 male, 104 female) were collected from the healthy individuals within the Jordanian population, aged 1 to 15. Healthy individuals were selected from those without a family history of T1D. Additionally, 200 samples were gathered from long term T1D patients (81 male, 119 female) within the same age range. All participants were originally from Jordan. The samples were collected by the cooperation of the Royal Medical Service Hospitals in Jordan. This study was approved by the Institutional Ethics Review Board of Near East University (YDU/2017/48–427). Informed consent was obtained from all participants. Each subject completed a questionnaire that collected personal information including age, gender, age of onset, ethnicity and family history.

### DNA extraction and genotyping

Genomic DNA was extracted following the manufacturer’s protocol using the Qiagen QIAamp DNA Mini Kit. The polymorphisms of HLA-DQA1, HLA-DQB1 and CTLA-4 genes were analyzed through the polymerase chain reaction-restriction fragment length polymorphism (PCR–RFLP) method, utilizing the primers from the study by Ongagna et al., which were verified using BLAST software [8]. PCR was performed in a total reaction volume of 50 µL, containing 2X Master Mix (Taq DNA polymerase, dNTPs, MgCl_2_, KCl and stabilizers) (New England Biolabs, United Kingdom), 1 nmol of reverse and forward primers (Burjuan, Jordan) and 0.2 µg DNA sample (Table 1). The PCR cycles included: initial denaturation at 95^o^C for 70 s, followed by 35 cycles of denaturation at 94^o^C for 30 s, annealing at 57^o^C for 30 s, extention at 72^o^C for 45 s, and a final extention at 72^o^C for 5 min. Agarose gel electrophoresis (2.5%) was performed to confirm the amplification of the genes. To detect HLA-DQA1 polymorphisms, Dde I, Fok I and Rsa I restriction enzymes were used. For HLA-DQB1 polymorphisms, Acy I, Hae III, Hha I and Hpa II restriction enzymes were applied. The CTLA-4 polymorphisms were analyzed using the restriction enzyme Bbv I (Table 1). All restriction enzymes were procured from New England Biolabs (United Kingdom). For the restriction digestion step, a mixture of12 samples was prepared by combining 156 µL nuclease free water, 12 µL restriction enzyme and 12 µL buffer. This mixture was then divided into 12 tubes. After adding 10 µL of PCR product to each tube, the samples were incubated at 36^o^C for 1 h and at 65^o^C for 20 min. Restriction digestion products were visualized by 3% agarose gel electrophoresis. Agarose gel (Cleaver Scientific, United Kingdom) was prepared by adding 2 µL of ethidium bromide (final concentration: 0.5 µg/mL), followed by solidification at room temperature for 20 min. Samples (10 µL from the restriction digestion step) were mixed with 2 µL of gel loding dye. Five µL 50 bp DNA ladder (size range: 50–1350 bp, New England Biolabs, United Kingdom) and 10 µL sample were loaded into the gel. The gel was run at 125 V for 50 min [8] (Supplementary file).

**Table 1:** Primers, restriction enzymes and expected band sizes for the detection of HLA-DQA1, HLA-DQB1 and CTLA-4 polymorphisms

Primer	Restriction Enzyme	Location	Target band size (bp)	Expected band size (bp)
5’-GGTGTAAACTTGTACCAG-3’ 5’-GGTAGCAGCGGTAGAGTTG-3’	Dde I Fok I Rsa I			225, 127, 118, 113 225, 187 225, 186, 183
		HLA-DQA1	225	
5’-GATTTCGTGTACCAGTTTAAG-3’ 5’-CCACCTCGTAGTTGTGTCTGC-3’	Acy I			241, 137, 104, 70
	Hae III	HLA-DQB1	241	241, 150, 127, 95
	Hha I			189, 141, 112, 90
	Hpa II			241, 198, 126, 115
5’-GCTCTACTTCCTGAAGACCT-3 5’-AGTCTCACTCACCTTTGCAG-3’	Bbv-I	CTLA-4	162	162, 88, 74

### Statistical analysis

Statistical analyses were done using MedCalc Statistical Software version 12.7.7 (MedCalc Software bvba, Ostend, Belgium; http://www.medcalc.org; 2013). Continuous variables are shown by descriptive statistics (average, standard deviation, minimum, median, maximum). Frequency and percentage values were calculated for categorical variables. Logistic regression analysis was used to find out Odds Ratio’s. Significance level was accepted as 0.05.

## Results

The demographic data are summarized in Table 2. In the control group, 48% of the subjects were male, 52% were female. In the patient group, percentages of the male and female individuals were 40.5% and 59.5%, respectively. No significant difference was found between groups in terms of gender. Among the 200 diabetic patients, 17% (*n* = 34) were between ages of 1–5, 43.5% (*n* = 87) were between 6 and 10, and 39.5% (*n* = 79) were between 11 and 15. A significant difference was observed in terms of age and family history, with patients aged 11–15 more likely to have a high rate of family history. The average age of onset was 5.9 ± 3.3 years. Gender, family history and age of onset did not correlate with allele frequencies.

**Table 2:** Description of study groups

	Control (*n* = 200)		Patient (*n* = 200)		
	Male	Female	Male	Female	*p*
Gender	96 (48%)	104 (52%)	81 (40.5%)	119 (59.5%)	0.159
Age (years)					< 0.001
1–5	58 (29%)		34 (17%)		
6–10	100 (50%)		87 (43.5%)		
11–15	42 (21%)		79 (39.5%)		
Age of onset (years)			5.9 ± 3.3 Med (Min-Max) 6 (1–13)		
Family History					< 0.001
Yes	0 (0%)		39 (19.5%)		
No	200 (100%)		161 (80.5%)		

The PCR-RFLP analysis of amplified DNA from 200 type 1 diabetic patients and 200 controls identified several HLA-DQA1 and HLA-DQB1 alleles associated with the disease (Tables 3 and 4). The alleles HLA-DQA1*01:01, HLA-DQA1*02:01 and HLA-DQB1*05:01 were found to be associated with T1D. Among these, only the HLA-DQA1*01:01 haplotype was identified as a risk factor for T1D. There was a statistically significant difference (OR 0.457, *p* < 0.001) in the occurence of HLA-DQA1*01:01 polymorphism since 49% of the diabetic patients have polymorphism in HLA-DQA1*01:01 whereas the percentage of the individuals having this haplotype in control group was 30.5%. Conversely, HLA-DQA1*02:01 (37.5% vs. 10.5%, OR 5.115, *p* < 0.001) and HLA-DQB1*05:01 (13.5% vs. 4%, OR 3.75, *p* < 0.001) haplotypes were significantly more prevalent in the control group than in the patient group, suggesting these haplotypes may offer protection against T1D. No significant association was found between CTLA-4 and T1D (Table 5).

**Table 3:** Prevalence of HLA-DQA1 polymorphisms in T1D and control groups

	Control		Patient		*p*	OR	95% CI for OR
	*N*	%	*N*	%			
DQA1*01:01	61	30.5%	98	49.0%	**< 0.001**	**0.457**	**0.30–0.69**
DQA1*01:02	17	8.5%	19	9.5%	0.727	0.885	0.45–1.75
DQA1*02:01	75	37.5%	21	10.5%	**< 0.001**	**5.114**	**2.99–8.73**
DQA1*03:01	11	5.5%	20	10.0%	0.097	0.524	0.24–1.12
DQA1*04:01	19	9.5%	23	11.5%	0.515	0.808	0.43–1.54
DQA1*05:01	16	8.0%	21	10.5%	0.390	0.741	0.38–1.47

**Table 4:** Prevalence of HLA-DQB1 polymorphisms in T1D and control groups

	Control		Patient		*p*	OR	95% CI for OR
	*N*	%	*N*	%			
DQB1*02:01	129	64.5%	146	73.0%	0.67	0.672	0.44–1.03
DQB1*03:02	23	11.5%	34	17.0%	0.118	0.634	0.36–1.12
DQB1*03:03	21	10.5%	12	6.0%	0.106	1.838	0.88–3.85
DQB1*05:01	27	13.5%	8	4.0%	**0.001**	**3.75**	**1.66–8.46**

**Table 5:** Prevalence of CTLA-4 polymorphisms in T1D and control groups

	Control		Patient		*p*	OR	95% CI for OR
	*N*	%	*N*	%			
CTLA-4 A/A	118	59.0%	125	62.5%	0.474	0.863	0.58–1.29
CTLA-4 G/G	25	12.5%	19	9.5%	0.339	1.361	0.72–2.56
CTLA-4 A/G	57	28.5%	56	28.0%	0.912	1.025	0.66–1.58

## Discussion

Type 1 diabestes (T1D) is an autoimmune disorder caused by the immune system attacking insulin-producing pancreatic β-cells. The disease is believed to develop when environmental triggers interact with a genetically susceptible individual. Among genetic factors, polymorphisms in HLA class II genes (located on chromosome 6p21.3) have the strongest link to T1D [13]. However, more than 50 other genes, primarily involved in immune function, have also been associated with the disease [14]. The complexity even increases with different genetic haplotypes [3]. Depending on the ethnic background, genetic variations can either protect against or contribute to T1D [15]. In Caucasian population, T1D is strongly linked to haplotypes such as DRB1*03:01-DQA1*05:01- DQB1*02:01 and DRB1*04:05- DQA1*03:01-DQB1*03:02, DRB1*04:01-DQA1*03:01-DQB*03:02, and DRB1*04:02-DQA1*03:01-DQB1*03:02 haplotypes, followed by the DRB1*04:04-DQA1*03:01-DQB1*03:02 and the DRB1*08:01-DQB1*04:01-DQB1*04:02 haplotypes. DRB1*15:01-DQA1*01:02-DQB1*06:02, DRB1*14:01-DQA1*01:01-DQB1*05:03 are considered as protective [16]. In contrast, DQA1*01:03, DQA1*02:01, DQA1*04:01, DQB1*03:01, DQB1*04:02, DQB1*05:01, DQB1*05:03, DQB1*06:01 and DQB1*06:02 alleles are considered protective in Chinese population [17]. In Iranian population, DRB1*04:05-DQB1*03:02, DRB1*04:02-DQB1*03:02 and DRB1*03:01-DQB1*02:01 haplotypes have been shown to have predisposing role in the development of T1D in children while DRB1*13:01-DQB1*06:03, DRB1*15:01-DQB1*06:02 were protective haplotypes [18]. Therefore, both ethnicity and geographical location should be taken into account when assessing HLA-DQA and DQB haplotypes in relation to T1D risk.

In our study, there was no difference in terms of gender between the two study groups. However, a notable difference was observed in age, with patients aged 11–15 more likely to have a family history of T1D. These findings align with previous research [12]. We also analyzed the relationship between the HLA-DQA1 and HLA-DQB1 allele frequencies and various demographic factors, including age of onset, gender and family history. Our results showed no significant association between these allele frequencies and examined demographic characteristics. Specifically, HLA-DQA1 and HLA-DQB1 alleles were similarly distributed across different age groups, genders and individuals with or without a family history of T1D. These findings indicate that while HLA-DQA1 and HLA-DQB1 alleles play crucial role in genetic susceptibility to T1D, they do not appear to have a direct relationship with the demographic characteristics assessed in this study.

The HLA-DQA1*01:01 allele was found to be significantly associated with T1D, with frequencies of 98 in the patient group and 61 in the control group, suggesting that this allele may serve as a potential risk factor for diabetes. However, in populations from Algeria [19] and Morocco [20], the same allele has been reported to have protective effect against T1D. In contrast, studies conducted in Sudanese [21], Italian (Lazio region) [22], and Greek [23] populations did not find any significant link between this allele and protection from the disesae.

HLA-DQA1*02:01 allele showed a significant difference between groups, with frequencies of 21 in the patient group and 75 in the control group, suggesting a potential protective role aganist T1D. Similarly, this genotype has been identified as protective in Moroccan [20], French and Spanish [6] and Italian [22] populations. However, studies in Sudanese [21] and Greek [23] populations did not find any significant association between HLA-DQA1*02:01 and protection against T1D.

HLA-DQB1*05:01 allele was observed to have a protective role against T1D, with frequencies of 8 in the patient group and 27 in the control group. Similar protective effects were reported in Moroccan [20] and Bahraini [24] populations. However, studies in Algerian [25] and Italian [22] populations did not find a significant correlation regarding its protective function.

Studies indicate that most HLA polymorphisms are linked to increased genetic susceptibility to T1D, though this varied accross ethnic groups [26]. In the Iranian population, HLA genotype has shown a correlation with age of onset and gender. The most prevalent haplotypes were DRB1*04:01, DQB1*03:02 and the HLA-DRB1*04:01-DQB1*03:02 alleles in men with T1DM. In contrast, females with T1DM were more likely to have the DRB1*03:01, DRB1*15:01, DQB1*06:01 alleles, DQB1*03:01/05:01 genotype, and DRB1*03:01–DQB1*02:01 and DRB1*15:01–DQB1*06:01 alleles [27]. In Southeast Asia and Japan, the DRB*04:01–DQB1*03:02 and DRB*03:01–DQB1*02:01 were less frequent, while DRB1*04:05–DQB1*04:01 and DRB1*09:01–DQB1*03:03 were the most prevalent HLA polymorphisms in Japan and Korea [28]. On the other hand, the most prevalent haplotype in Arab populations (Bahrain, Lebanon, and Tunisia) was DRB1*03:01–DQB1*02:01 [29]. In African-American population, individuals carrying DRB1*07:01, DRB1*03:03, and DQA1*03:01–DQB1*02:01 were more susceptible to T1D. Conversely, in European population HLA-DRB1*07:01–DQA1*02:01–DQB1*02:01 haplotype was a protective factor against T1D [30].

Beyond genetic predisposition, gut microbiota dysbiosis has recently been recognized as a potential risk factor for T1D due to similarities between gut bacterial autoantigens and pancreatic islet cells. Shirizadeh et al. found that the DRB1*03:01, DQB1*02:01 and DQB1*03:02 allels and the DRB1*03:01 ~ DQB1*02:01 haplotype were more frequent in T1D patients compared to their parents and siblings. In contrast, DRB1*11:01, DRB1*14:01 and DQB1*03:01 alleles were significanly lower. Additionally, DRB1*04 ~ DQB1*03:02 haplotype was linked with the development of glutamic acid decarboxylase antibody (GADA) and insulinoma-associated antigen-2 antibody (IA-2 A), whereas DRB1*11:01 ~ DQB1*03:01 was associated with seronegativity [31].

In the present study, CTLA-4 A/A (frequencies were 125, 118 in patient and control groups, respectively), A/G (frequencies were 56, 57 in patient and control samples respectively) and G/G (frequencies were 19, 25 in patient and control groups, respectively) showed no significant correlation with T1D, same results were also observed in Jordanian population [9]. However, in Sudanese population, a significant difference was reported in both genotype and allele frequencies of the CTLA-4 (+ 49 A/G) polymorphism between the patients and the controls. Specifically, patients had significantly higher frequencies of the G allele, GG homozygous genotype, and AG heterozygous genotype than controls [32]. This finding was also consistent with the polymorphism study conducted in Ethiopian population. An association between the G allele and T1D was discovered and particularly a correlation between the GG genotype of the CTLA-4 (+ 49 A/G) gene polymorphism and the tendency of having T1D was found [33]. These findings were in contrast to research conducted in Brazil [34], Chili [35], Turkey [36], and Egypt [37], which suggested that the CTLA-4 (+ 49 A/G) polymorphism was not identified as a risk factor for type 1 diabetes. The inconsistency in results could be explained by the small sample size, genetic heterogeneity in the studied populations, and the various environmental factors involved in the pathogenesis of T1D.

## Conclusion

Only the HLA-DQA1*01:01 haplotype has shown correlation in terms of T1D risk among all the haplotypes examined. However, it has been discovered that two of them, HLA-DQA1*02:01 and HLA-DQB1*05:01, are protective against T1D. The A/A, A/G, and G/G CTLA-4 genotypes did not differ between the control and T1D groups. Although our analysis focused on association between T1D and haplotypes, these findings provide valuable insights into genetic predisposition to diabetes. We recommend the integration of screening programs to identify individuals at higher risk. Awareness could enable personalized intervention strategies, such as immunomodulatory approaches, continuous clinical monitoring and life style modifications. In addition, public health initiatives aimed at increasing awareness of genetic risk factors could contribute to the prevention efforts.

### Study limitations

While this study provides valuable insights into the genetic factors associated with Type 1 Diabetes (T1D) in the Jordanian population, there are several limitations that must be considered.

1. One of the key limitations of this study is the exclusion of HLA-DR alleles from the analysis. HLA-DR alleles, in addition to HLA-DQ alleles, are well-established genetic markers for susceptibility to T1D, playing a crucial role in the immune system’s response to autoantigens. Including HLA-DR alleles would have provided a more comprehensive understanding of the genetic risk factors for T1D in the Jordanian population. Future studies should address this gap by investigating the role of HLA-DR alleles in conjunction with HLA-DQ alleles to offer a more thorough assessment of genetic predisposition.
2. Although the sample size in this study was sufficient for detecting broad trends, it may not have been large enough to capture rare allele variations or genetic differences within smaller subgroups of the Jordanian population. This may limit the generalizability of the findings, particularly when it comes to understanding genetic risk in specific demographic groups, such as those with early or late-onset T1D.
3. Our study focused solely on genetic markers, particularly HLA-DQA and HLA-DQB alleles, and did not account for potential environmental or lifestyle factors that could interact with these genetic factors to influence the risk of T1D. Future studies should incorporate a more comprehensive approach that considers the interplay between genetics and environmental/lifestyle factors (diet, infections etc.) to better understand the multifactorial nature of T1D.

## Electronic supplementary material

Below is the link to the electronic supplementary material.

Supplementary Material 1

## Data Availability

No datasets were generated or analysed during the current study.

## Declarations

### Authors note

This article is a revised version of the Master’s thesis titled “HLA Class II Polymorphisms as a Risk Factor in Jordanian Patients with Type I Diabetes Mellitus” completed by Monther Hussain Obaied at Near East University (http://org.library.neu.edu.tr/cgi-bin/koha/opac-detail.pl?bib=285988).
