# Supplementary material for: Implication of the HLA-DQA1, HLA-DQB1 and CTLA-4 alleles in the susceptibility to type 1 diabetes in Jordanian population
Source: Mol Biol Rep. 2025 Mar 21;52(1):330. doi: 10.1007/s11033-025-10438-x (PMC11928406; doi:10.1007/s11033-025-10438-x)
Supplement: Supplementary file 1 — Supplementary Material 1 [file 11033_2025_10438_MOESM1_ESM.docx]

Supplementary Figure 1. Agarose gel electrophoresis (3%) of restriction digestion products of HLA-DQA1, HLA-DQB1 and CTLA-4 haplotypes.
